# Drum Communication Program Intervention in Older Adults With Cognitive Impairment and Dementia at Nursing Home: Preliminary Evidence From Pilot Randomized Controlled Trial

**DOI:** 10.3389/fnagi.2020.00142

**Published:** 2020-07-02

**Authors:** Atsuko Miyazaki, Takashi Okuyama, Hayato Mori, Kazuhisa Sato, Masahiko Ichiki, Rui Nouchi

**Affiliations:** ^1^Computational Engineering Applications Unit, Head Office for Information Systems and Cybersecurity, RIKEN, Saitama, Japan; ^2^Department of Physical Therapy, Faculty of Health Sciences, School of Medicine, Kobe University, Kobe, Japan; ^3^Technology and Innovation Hub, Cluster for Science, RIKEN, Saitama, Japan; ^4^Medical Collaboration Division, Care 21 Co., Ltd., Tokyo, Japan; ^5^Department of Psychiatry and Behavioral Sciences, Tokyo Medical University, Tokyo, Japan; ^6^Department of Cognitive Health Science, Institute of Development, Aging and Cancer (IDAC), Tohoku University, Sendai, Japan

**Keywords:** drumming communication program, older adults, nursing homes, cognitive functions, physical functions, randomized controlled trial

## Abstract

**Introduction**: Inactivity and consequent deterioration of cognitive and physical function is a major concern among older adults with the limited walking ability and need a high level of care in nursing homes. We aimed to test whether a drumming communication program (DCP) that uses the rhythmic response function of the elderly with cognitive impairment, dementia, and other debilitating disorders would improve their cognitive and physical function.

**Methods**: We conducted a Randomized Controlled Trial (RCT) to investigate the effects of the DCP in 46 nursing home residents who needed high levels of nursing care. The participants were randomly assigned to an intervention and control group. The intervention group attended 30 min of the DCP thrice a week for 3 months. Cognitive function was measured using the Mini-Mental State Examination-Japanese (MMSE-J) and Frontal Assessment Battery (FAB). Physical function was measured using grip strength and active upper limb range of motion with the dominant hand. Body composition was measured using bioelectrical impedance analysis (BIA). These measures were analyzed before and after the DCP intervention period, and data for the two groups were compared thereafter.

**Results**: Initially, the participants had low scores on the MMSE-J, and 84.78% of them used wheelchairs. Following the DCP intervention, the MMSE-J and FAB scores of the DCP group improved significantly. In terms of motor function, the active range of motion of the wrist palmar and the shoulder flexion improved in the intervention group. Regarding body composition, the skeletal muscle mass index, total body protein, and the dominant hand muscle mass that was adding physical load decreased.

**Conclusions**: The DCP provided the participants with an opportunity to engage in continued exercise for 3 months. The intervention group exhibited improved cognitive function and upper limb motion range, and changes in body composition. The results suggest that DCP can be used as an intervention method to promote exercise and improve various health and cognitive functions.

**Trial Registration**: This trial was registered at the University Hospital Medical Information Network Clinical Trial Registry (UMIN000024714) on 4 November 2016. The URL is available at https://upload.umin.ac.jp/cgi-open-bin/ctr_e/ctr_view.cgi?recptno=R000028399.

## Introduction

Nursing homes cater to the elderly who require a high level of care; thus, they play an important role in the care of the elderly who are at their end-of-life. Among the residents of nursing homes in Japan, 96.7% had dementia, 39% were over 90 years of age, and 61.8% were bedridden, with dementia that required high levels of care (Ministry of Health, Labour and Welfare, 2016).

Low activity among nursing home residents is one of the major issues that nursing homes face. Nursing home residents spend up to 65% of their time being inert and doing nothing (Ice, 2002; den Ouden et al., 2015). Moreover, previous studies have demonstrated that they have a low physical work capacity (Stamford, 1972, 1973) and low hand motor function (Ostwald et al., 1989). Many previous studies have been concerned with difficulties in activities of daily living (ADL). Rapid increases in physical impairment (Banaszak-Holl et al., 2011) and the worsening of ADL dependence were found to occur in long-term nursing home residents (McConnell et al., 2002; Burge et al., 2013; Jerez-Roig et al., 2017).

Exercise therapy and exercise programs require participants to perform imitation mechanisms. However, patients with dementia have difficulty comprehending commands or imitating gestures (Vuorinen et al., 2000; Wheaton and Hallett, 2007). In addition to memory disorder, patients may also experience visuospatial analysis impairment (Rousseaux et al., 2012) and imitation gesture impairment (Nagahama et al., 2015; Li et al., 2016). In patients with all types of dementia, several kinds of apraxia are seen in the early stages of the disease (Chandra et al., 2015). Apraxias are motor dysfunctions that occur despite the absence of motor paralysis and include elementary motor, sensory deficit, or language comprehension disorders (Liepmann, 1900, 1905). As walking ability remains relatively separate from apraxia, the phenomenon of wandering behavior in patients with dementia poses a risk (Algase et al., 2007). Previous studies that used exercise programs on dementia patients have reported on walking-based erobic type physical activities (Öhman et al., 2014; Forbes et al., 2015). Such studies show positive motor and cognitive effects (Santana-Sosa et al., 2008; Frederiksen et al., 2014). However, walking training is difficult in nursing homes with elderly patients require high levels of care. Therefore, our drumming communication program (DCP) is designed for the needs of patients with dementia who have difficulty exercising or walking in a nursing home.

Previous studies have indicated that the rhythmic function of patients with severe dementia is not significantly different from that of healthy elderly people, regardless of whether the former have Alzheimer’s disease (AD) or other forms of dementia (York, 1994, 2000). For dementia patients, it is predicted that exercise can be induced by utilizing their retained ability to identify rhythm in music (Vasionyte and Madison, 2013), such as in drum performance. When a person plays the drum, they only need to hit the drum with a bouncing mallet, which is simple and easy. Regarding the interaction between the drum and the player, the mallet has been found to rebound as it radiates much of the drum’s impact energy and elastically returns the energy to the player. If the player learns how to use the rebound, he or she can get a sound with very little effort, even if he or she was a beginner (Fujisawa and Miura, 2010).

Drumming is an exercise (Amad et al., 2016) that can generate the positive effects of cardiovascular exercise (De La Rue et al., 2013). It is known that entrainment by the beat of the music has a stimulating effect on motor circuits at the neuronal functioning level (Hove et al., 2007; Thaut et al., 2015; Burger et al., 2018). This finding was confirmed in a patient with Parkinson’s disease whose walking improved; by merely listening to rhythm stimulation, his walking corresponded to the beat of the music (Thaut et al., 1996; Hausdorff et al., 2007). Rhythmic auditory stimulation (RAS) induces brain plasticity in a damaged brain by rhythmic entrainment, which is called neurological rehabilitation (Thaut and Abiru, 2010). RAS also has beneficial effects for other neurological diseases or brain injuries, as the synchronization to an external beat helps recover the coordination of movements (Bradt et al., 2010; Rodriguez-Fornells et al., 2012; Miendlarzewska and Trost, 2014).

Additionally, Rhythm Synchronization Tasks using tapping are related to various cognitive abilities that include not only listening skills, executive function, and auditory processing, but also working memory, spatial cognition, and linguistic and perceptual skills (Tierney and Kraus, 2013). Although previous studies have presented the existence of a sensitive period hypothesis for musical training (Trombetti et al., 2011), exercise programs using rhythm response have also been described as improving with training, even for those who are older than 65 years of age (Rabinowitz and Lavner, 2014) and those with Parkinson’s disease who have passed the critical period (Bella et al., 2017). A recent study demonstrated that drum training is related to an increase in the neural plasticity of the brain structure, and that drum training for exercise-based intervention could potentially benefit rehabilitation treatment by overcoming impairments due to brain diseases (Amad et al., 2016).

While physical activity can be an important improvement factor in cognitive decline and dementia, the severity of the dementia of nursing home residents may prevent them from obtaining the effects of exercise and functional training. The nature of rhythmic entrainment through collective drumming can support increased opportunities and motor activities for exercise, even for patients with dementia and other diseases. In this study, we developed a DCP that uses the ability of patients with severe dementia to identify the rhythm of music and exercise with rhythmic responses.

**Figure 1:**
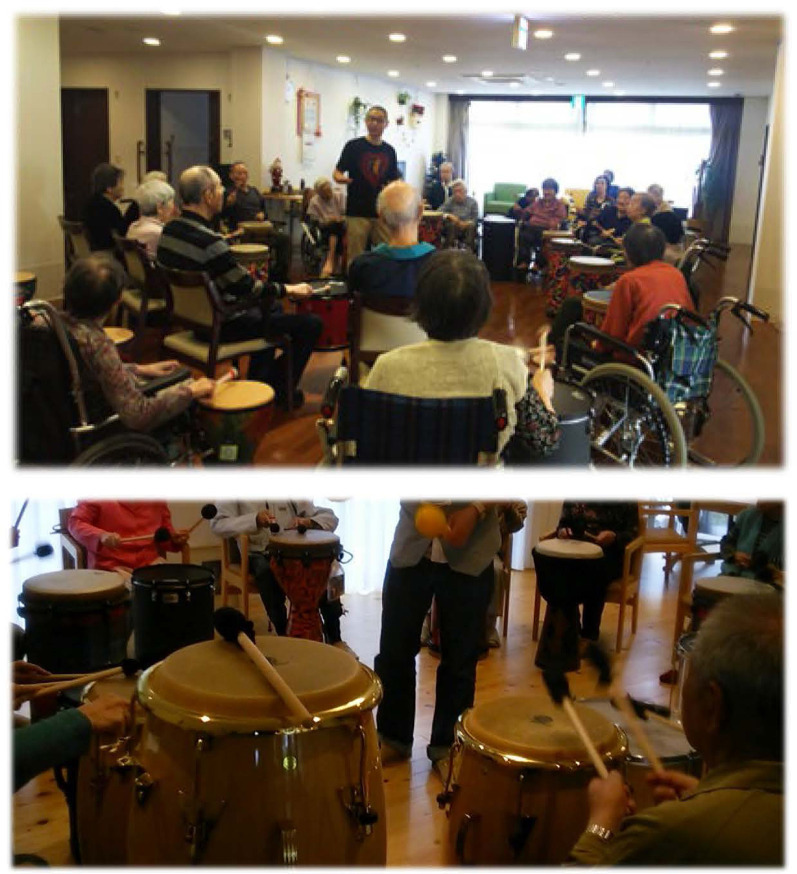
Drums for the drumming communication program (DCP) intervention.

We distributed various drum types to all participants in the drum circle, such as the djembe, tam-tam, tubano, tan-tan, and bahia (Ragg et al., 2019; Figure 1). Participants who could not reach their percussion instrument were provided a lightweight frame drum on the wheelchair table. During the program, all of the participants were seated in a circle and played the drum with a mallet. Participants only needed to hit the drum when a facilitator instructed them to (Hull, 2007). As a consequence of this study, we propose a rehabilitation program based on playing the drum to improve the cognitive and motor functions of patients with dementia and other debilitating diseases that have rhythm response capability.

The program rationale is as follows: (1) physical activity is beneficial for patients with mild, moderate, and severe dementia (Blankevoort et al., 2010). Studies on physical activity have focused on the training of lower limbs, measured by walking ability and gait speed. Unfortunately, in the present study, 84.78% of the participants use wheelchairs. Therefore, gait and lower limb training are impossible. Moreover, participants with dementia have difficulty imitating the commands and gestures necessary for exercise therapy and programs (Vuorinen et al., 2000; Wheaton and Hallett, 2007). The participants of the present study scored lower on the Mini-Mental State Examination (MMSE) than participants of previous studies at baseline. It was difficult for them to move their upper limbs according to the instructions for measuring grip strength. It was also difficult for them to understand and to learn new exercises. (2) However, the rhythm response function is maintained in patients with severe dementia (York, 1994, 2000). This makes it possible to accurately perceive timing information and generate reactions or motions related to timing (Thaut, 2003). Simple drum playing causes repeated upper limb motion. Repetitive motion through physical rehabilitation promotes muscle flexibility, improved range of motion, increases muscle mass, and strengthens exercise tolerance (Kisner and Colby, 2007; Langhorne et al., 2009). Therefore, the rhythm response function in playing drums can be used to create a new exercise program for people with dementia and other debilitating disorders. (3) Previous studies have found that there is a bilateral correlation between physical function and cognitive function (Laurin et al., 2001; Rockwood and Middleton, 2007; Rockwood et al., 2007; Auyeung et al., 2008). Exercise and physical activity not only increase cardiorespiratory function and muscle mass but also have beneficial effects on the brain by regulating neurotrophins (Raichlen et al., 2019). For example, exercise increases the production of brain-derived neurotrophic factor (BDNF), resulting in the generation of new neurons and improved connectivity between existing neurons (Neeper et al., 1995). These exercise-induced neuroplasticities have also been shown to improve brain volume, memory, and executive function, even in Randomized Controlled Trial (RCT)s on participants with AD or at risk for AD (Colcombe et al., 2006; Nascimento et al., 2014; Cheng, 2016; Gaitán et al., 2019). Unsupported and free upper limb movements, such as drumming movements, also result in the training of muscles that increase the weight of the upper limb and support the same (Ries et al., 2007). It has also been suggested that drumming may have been involved in the generation of brain plasticity due to physical activity.

We hypothesize that cognitive function will improve when there is an exercise effect or physical function improvement *via* drumming. Thus, the purpose of the present study is to evaluate the benefits of the newly developed DCP program on the cognitive and physical function in older adults with cognitive impairment and dementia in a nursing home.

## Materials and Methods

### Randomized Controlled Trial Design and Trial Setting

This RCT was conducted between December 2016 and March 2017 in a special care nursing home for the elderly in Tokorozawa, Saitama, Japan. Given that the participants could not directly understand the purpose of the study, we provided an adequate explanation of the trial to the participants’ families. Those who agreed to participate provided written informed consent. The Ethics Committee of RIKEN approved this study (ref. Wako327-12). Moreover, the study was conducted following the Declaration of Helsinki (1991) and was registered in the University Hospital Medical Information Network (UMIN) Clinical Trial Registry (UMIN000024714).

We conducted an open RCT; no one was blind to the study because whoever was playing the drum was apparent, with two groups: a DCP group and a control (no treatment) group. Although this study was an open RCT, the control group never saw the other group playing drums or knew about the program. Research personnel who administered cognitive and physical function tests were blind to the group assignment as well.

**Figure 2:**
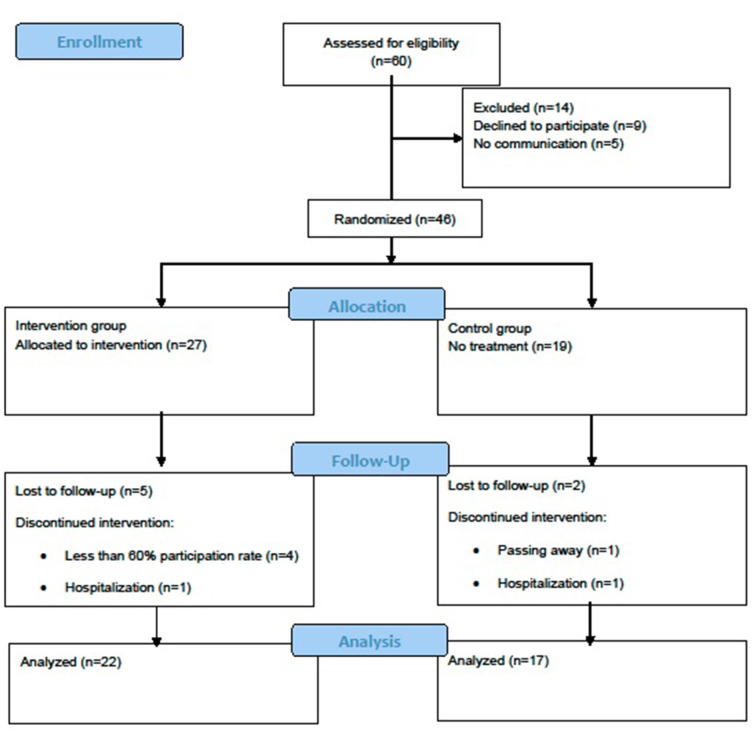
Consolidated standards of reporting trial (CONSORT) flow diagram.

The Consolidated Standards of Reporting Trials (CONSORT) statement was used to report the study structure (Schulz et al., 2010). The RCT design is presented in Figure 2.

### Randomization

Using their total Mini-Mental State Examination-Japanese (MMSE-J; Sugishita et al., 2018) scores, a psychiatrist divided the participants into four categories: those with severe dementia (0–10 scores), moderate dementia (11–20 scores), mild dementia (21–26 scores), and no dementia (27–30 scores; Perneczky et al., 2006). Since the highest total MMSE-J score was 27, the participants were divided into three categories. Then, they were randomly assigned to the DCP group (intervention group) or control group (no treatment group) using dynamic allocation in the order that the MMSE-J was measured.

### Participants

Sixty participants were recruited from the residents of the special nursing home (out of 100 residents) in Tokorozawa, Saitama, Japan. A special nursing home in Japan is a facility that provides the highest level of care for older adults outside of a hospital, in a long-term stay toward the end of their lives. Participant recruitment procedures were briefing sessions for the staff at an elderly nursing home. The staff then recruited applicants from the pool of residents.

The inclusion criteria were an interest in participating in the study, and the ability to remain in a sitting position in a wheelchair for 30 min. Nine individuals’ families declined participation. The exclusion criteria consisted of those applicants who were deemed by a psychiatrist to be unable to answer questionnaires. Based on the results of the screening from the psychiatrist, five residents were found to be unable to respond to questionnaires. The remaining number of participants was 46 (men = 6; women = 40; average age = 87.04, SD = 6.72). The average length of stay in a nursing home was 801.20 days (SD = 602.54). The independence in daily living for older persons with dementia (ID-ADL) was assessed using the scale provided by the Ministry of Health, Labour, and Welfare of Japan, which is an observer-based rating scale and is consistently used in long-term care insurance systems of Japan. This scale includes seven categories (I, IIa, IIb, IIIa, IIIb, IV, M), with a higher score indicating more severe dementia. Regarding the ID-ADL scale of the participants, I = 1, IIa = 9, IIb = 19, IIIa = 10, IIIb = 4, IV=3, and *M* = 0 persons.

### Sample Size

The sample size was not predetermined for this pilot study.

### Overview of Intervention

For the intervention group, the 30-min DCP program was conducted three times per week for 3 months (36 sessions total). They were split into two groups, and the sessions were conducted consecutively in a common area of the nursing home. The staff took a roll call for the intervention group participants (27), who had to attend a minimum of 60% of the sessions to be included in analysis. The control group had no intervention and no treatment. For 3 months, they continued with their daily lives as usual. The control group never knew about the drum sessions because they were resting in another room on a different floor. Therefore, they were blind to the DCP intervention.

Assessments of cognitive and physical function were carried out before and after the intervention. Both groups of participants also completed a series of neuropsychological and physical tests before beginning the program (pre). There were no significant differences between the groups in those tests (two-sample *t*-test, *p* > 0.10; Table 1). After 3 months, both groups of participants were reexamined using the same tests (post). After finishing the tests, the members of the control group participated in a one-time session of DCP.

**Table 1:** Baseline data for drumming communication program (DCP) intervention and control (no-treatment) groups.

Criterion	Intervention group (*n* = 27)		Control group (*n* = 19)		*T*-test
	Mean (SD)	Range	Mean (SD)	Range	*p*-value
Age, years	85.74 (6.96)	72–101	88.89 (6.05)	78–100	0.12
Sex (female:male)	22:5		18:1		
Length of stay (days)	706.89 (570.10)	74–1730	935.16 (637.09)	103–1742	0.21
ID-ADL (7categories)	3.30 (0.99)	1–6	3.42 (1.39)	2–6	0.72
**Cognitive function measures**					
MMSE-J total score	12.93 (6.64)	0–23	12.89 (6.89)	0–25	0.99
Orientation	2.85 (2.88)	0–9	2.53 (2.39)	0–7	0.69
Registration	2.19 (1.14)	0–3	2.37 (1.01)	0–3	0.58
Spell backwards in reverse	1.00 (1.64)	0–5	1.05 (1.65)	0–5	0.92
Recall	0.37 (0.63)	0–2	0.74 (1.05)	0–3	0.15
Language: names	1.70 (0.67)	0–2	1.37 (0.90)	0–2	0.15
Language: repeat	0.93 (0.38)	0–2	0.74 (0.45)	0–1	0.13
Language: 3 stage command	2.59 (1.01)	0–3	2.74 (0.81)	0–3	0.61
Language: following a written command	0.70 (0.47)	0–1	0.68 (0.48)	0–1	0.89
Language: writing a sentence	0.33 (0.48)	0–1	0.26 (0.45)	0–1	0.62
Visuospatial abilities: copying a diagram	0.26 (0.45)	0–1	0.42 (0.51)	0–1	0.26
FAB total score	6.37 (3.12)	3–13	6.21 (3.75)	0–13	0.88
Similarities (conceptualization)	0.67 (0.83)	0–3	0.47 (0.70)	0–2	0.41
Lexical fluency (mental flexibility)	0.96 (1.09)	0–3	0.68 (1.06)	0–3	0.39
Motor series (programming)	0.78 (0.80)	0–3	1.16 (1.17)	0–3	0.20
Conflicting instructions (sensitivity to interference)	0.81 (1.11)	0–3	0.84 (1.12)	0–3	0.94
Go–No Go (inhibitory control)	0.37 (0.63)	0–2	0.32 (0.48)	0–1	0.75
Prehension behavior (environmental autonomy)	0.81 (1.11)	0–3	0.84 (1.12)	0–3	0.94
**Mood state measures**					
GDS (Score)	6.41 (3.12)	1–15	6.26 (2.64)	2–11	0.87
LSI-K (Score)	5.67 (2.00)	2–9	5.00 (1.67)	2–7	0.24
**Motor function measures**					
Hand grip strength (in kg)	9.87 (5.95)	0.00–24.50	7.03 (4.96)	0.00–14.30	0.10
Active shoulder flexion (°)	119.26 (28.78)	70–180	107.89 (25.29)	50–145	0.17
Active elbow flexion (°)	143.70 (3.56)	130–145	141.05 (6.99)	125–145	0.10
Active elbow extension (°)	−10.56 (15.65)	−40 to 10	−14.74 (10.20)	−30 to 0	0.31
Active wrist palmar flexion (°)	50.37 (19.51)	5–90	40.53 (16.66)	10–70	0.08
Active wrist dorsi flexion (°)	48.15 (23.54)	−45 to 70	40.79 (12.72)	25–60	0.22
**Body composition measures**					
Body height (cm)	148.07 (9.74)	128.00–174.00	147.32 (5.82)	138.50–160.04	0.77
Body weight (kg)	45.16 (9.87)	27.80–64.10	42.19 (7.23)	30.00–53.00	0.27
Body muscle mass (kg)	29.37 (5.99)	21.00–46.40	28.55 (5.13)	21.10–40.20	0.63
Body ECW/TBW	0.41 (0.01)	0.40–0.43	0.41 (0.01)	0.40–0.43	0.84
Protein (kg)	5.89 (1.23)	4.10–9.50	5.71 (1.02)	4.20–7.90	0.60
BMI (kg/m^2^)	20.51 (3.72)	13.90–30.20	19.39 (2.89)	14.20–23.40	0.28
SMI (kg/m^2^)	5.01 (1.02)	3.15–7.18	4.61 (1.21)	2.80–7.12	0.24
Dominant hand muscle mass (kg)	1.34 (0.47)	0.72–2.60	1.24 (0.35)	0.74–1.95	0.42
Dominant hand ECW/TBW	0.39 (0.01)	0.37–0.41	0.39 (0.01)	0.38–0.40	0.25
Arm SMM (kg/m^2^)	1.22 (0.34)	0.65–2.07	1.13 (0.30)	0.67–1.78	0.36

*Values given are mean (standard deviation) unless stated otherwise. MMSE-J, Mini-Mental State Examination-Japanese; FAB, Frontal Assessment Battery at Bedside; GDS, Geriatric Depression Scale; LSI-K, Life Satisfaction Index-K; Body ECW/TBW, body extracellular water ratio; Protein, total body protein; BMI, body mass index; SMI, skeletal muscle mass index; Arm SMM; arm skeletal muscle mass*.

### DCP Group (Intervention Group)

The program was guided by a facilitator with experience in leading community drum circle activities (Hull, 2007). The facilitator was a drummer and certified drum instructor for over 20 years. The participants’ connection and communication skills were strengthened as a result of the DCP. The participants, who attended to enjoy the experience and not to learn how to play the drums, picked up the beat and played whatever they wanted. The facilitator occasionally guided them to shift to a strong or soft rhythm, slow or fast tempo, or small or big beats. He also led them through various “call-and-response” exercises, where they copied rhythms, as well as “stop-and-cut” exercises, where they had to determine the proper time to stop and start. The facilitator also emphasized improvization. The participants played rhythms freely to create their performances and experience listening to others’. The facilitator gradually increased the complexity of the rhythms, but only made such modifications after ascertaining the participants’ comfort with the difficulty level. Instead of teaching the participants, the facilitator simply provided instructions and generated an atmosphere of improvisatory drumming along with musical accompaniment based on the participants’ abilities.

The facilitator was not a music therapist, did not know the details of participants’ backgrounds or their psychological profiles, and was not aware of the specific psychological aims of the DCP program. Therefore, he was likewise blind to the purpose of the study.

Most of the participants were using wheelchairs and could not place a drum in between their legs. Because the drum was installed within the reach of the mallet on their dominant hand side, they drummed only with their dominant hand (Figure 1).

### Control Group (No Treatment Group)

The control group consisted of 19 participants who did not attend the DCP during the 3 months. These participants were informed that they were scheduled to receive an invitation to participate in a one-time DCP session after a waiting period of 3 months. In the meantime, they continued with their lives as usual, without changing their schedule for the duration of the study. While the nursing home had other music programs, such as karaoke, they had no opportunities to play the drum.

The below sections discuss the cognitive and physical function measures. The participants’ baseline characteristics are presented in Table 1.

### Cognitive Function Measures

Participants’ cognitive functions were tested using the following screening instruments: the MMSE-J (Sugishita et al., 2018) and the Frontal Assessment Battery (FAB; Dubois et al., 2000). The MMSE-J is the Japanese version of the MMSE (Folstein et al., 1983); it is a 30-item instrument used to screen for dementia. The items in MMSE measure Orientation, Registration, Attention and Calculation, Recall, Language: names, Language: repeat, Language: 3-stage command, Language: following a written command, Language: writing a sentence, and Visuospatial abilities: copying a diagram. The “Attention and Calculation” aspect comprises two types of tasks: serial calculation and the “spell ‘w-o-r-l-d’ in reverse” test in the English version. We chose the “spell backward in reverse” test in MMSE-J (Table 2). Specifically, we asked the participants to pronounce the syllable “se-ka-i-chi-zu” backward.

**Table 2:** Comparison of score changes between the intervention and control groups following the DCP intervention.

	Intervention group (*n* = 22)		Control group (*n* = 17)		Permutation test *p*-value	Adjusted by FDR *p*-value		Effect size (*η*^2^)
**Cognitive function measures**
MMSE-J total score	2.05	3.98	−3.24	4.87	0.000	0.004*	DCP > NT	0.36
Orientation	0.50	1.95	−0.53	1.77	0.015	0.075	ns	0.12
Registration	0.32	0.95	−0.47	1.28	0.033	0.121	ns	0.10
Spell backward in reverse	0.05	1.70	−1.06	1.52	0.002	0.018*	DCP > NT	0.27
Recall	0.50	0.96	−0.41	0.94	0.007	0.047*	DCP > NT	0.15
Language: names	0.05	0.90	−0.35	0.86	0.011	0.060	ns	0.14
Language: repeat	0.09	0.43	0.12	0.78	0.363	0.892	ns	0.01
Language: 3 stage command	0.41	1.05	−0.47	1.50	0.002	0.028*	DCP > NT	0.19
Language: following a written command	0.14	0.47	0.00	0.35	0.204	0.621	ns	0.03
Language: writing a sentence	0.00	0.53	−0.18	0.53	0.057	0.199	ns	0.10
Visuospatial abilities: copying a diagram	0.00	0.62	−0.06	0.43	0.382	0.892	ns	0.00
FAB total score	2.36	3.35	−0.35	2.15	0.006	0.043*	DCP > NT	0.19
Similarities (conceptualization)	1.05	1.25	0.76	1.15	0.373	0.892	ns	0.01
Lexical fluency (mental flexibility)	0.05	0.58	0.06	0.97	0.333	0.864	ns	0.02
Motor series (programming)	0.55	1.44	−0.35	1.17	0.029	0.111	ns	0.09
Conflicting instructions (sensitivity to interference)	0.14	0.99	−0.47	0.94	0.010	0.060	ns	0.16
Go–No Go (inhibitory control)	0.41	1.10	−0.18	0.39	0.006	0.043*	DCP > NT	0.17
Prehension behavior (environmental autonomy)	0.23	0.87	0.00	0.71	0.330	0.864	ns	0.00
**Mood state measures**
GDS (Score)	−0.41	2.87	0.06	3.03	0.297	0.864	ns	0.02
LSI-K (Score)	0.32	2.44	0.18	1.88	0.101	0.336	ns	0.05
**Motor function measures**
Hand grip strength (in kg)	0.66	2.62	0.44	2.51	0.114	0.363	ns	0.04
Active shoulder flexion (°)	6.14	28.37	−5.88	14.92	0.004	0.040*	DCP > NT	0.15
Active elbow flexion (°)	−1.82	8.10	−6.47	21.85	0.500	1.000	ns	0.01
Active elbow extension (°)	3.41	14.01	2.35	11.06	0.490	1.000	ns	0.02
Active wrist palmar flexion (°)	10.91	35.17	−1.47	11.56	0.000	0.000*	DCP > NT	0.33
Active wrist dorsiflexion (°)	4.55	17.92	3.53	11.96	0.074	0.083	ns	0.11
**Body composition measures**
Body weight (kg)	−0.38	2.11	−0.34	2.28	0.480	1.000	ns	0.00
Body muscle mass (kg)	−0.57	1.83	0.59	1.25	0.018	0.083	ns	0.22
Body ECW/TBW	0.00	0.01	0.00	0.01	0.412	0.930	ns	0.01
Protein (kg)	−0.10	0.37	0.14	0.27	0.002	0.028*	DCP < NT	0.20
BMI (kg/m^2^)	−0.14	1.03	−0.22	1.22	0.461	1.000	ns	0.00
SMI (kg/m^2^)	−0.20	0.46	0.10	0.32	0.002	0.028*	DCP < NT	0.21
Dominant hand muscle mass (kg)	0.00	0.13	0.08	0.18	0.005	0.042*	DCP < NT	0.17
Dominant hand ECW/TBW	0.00	0.01	0.00	0.01	0.319	0.864	ns	0.05
Arm SMM (kg/m^2^)	−0.01	0.13	0.05	0.14	0.022	0.089	ns	0.14

*p < 0.05. SD, standard deviation; Score changes, post score minus pre score; MMSE-J, Mini-Mental State Examination-Japanese; FAB, Frontal Assessment Battery at Bedside; GDS, Geriatric Depression Scale; LSI-K, Life Satisfaction Index-K; Body ECW/TBW, body extracellular water ratio; Protein, total body protein; BMI, body mass index; SMI, skeletal muscle mass index; Arm SMM; arm skeletal muscle mass; NT, no treatment; ns, not significant.

The FAB is an 18-point instrument presented on a screen used to assess executive cognitive functions. FAB items include Similarities (conceptualization); Lexical Fluency (mental flexibility); Motor Series (programming); Conflicting Instructions (sensitivity to interference); Go No-Go (inhibitory control); and Prehension Behavior (environmental autonomy).

### Mood State Measures

Before the intervention period, we used questionnaires to assess the participants’ mood states: namely, the Geriatric Depression Scale short form (GDS-15; Brink et al., 1982), and the Life Satisfaction Index-K (LSI-K; Koyano, 1993).

The GDS-15 Japanese version (Sugishita et al., 2017) has been used to assess the symptoms of depression in a variety of older adults. The GDS-15 is a 15-item questionnaire that can be administered through an interview. The responses are provided in a “yes or no” format to be easy to understand for older people who have impaired cognitive function. The total score ranges from 0 to 15 with a cut-off of five, with a higher score reflecting more depressive symptoms.

LSI-K was used to assess life satisfaction through variables of subjective well-being. The LSI-K includes nine items and can be administered through an interview. A positive selection earns 1 point, with other options having no points. The life satisfaction score ranges from 0 to 9, with a lower score indicating poor quality of life.

### Motor Function Measures

We measured motor function abilities using grip strength and upper limb range of motion with the dominant hand with the aid of a physiotherapist.

Maximum grip strength of the dominant hand (in kg) was measured using the digital dynamometer grip (Takei D T.K.K.5401, Takei Scientific Instruments, Tokyo, Japan). The participants were asked to perform two or more maximum force trials with their dominant hand to obtain an accurate number. The instrument could not measure a rating of 5 kg or less; when the maximum values of the dominant hand were 5 kg or less, the measured value was 0. The physiotherapist confirmed through a handshake whether the participant had clearly scored 5 kg or less.

For the active movement of the upper limb, we measured the motor function at the main joints. The active range of motion measurements were taken for five upper-extremity motions: shoulder flexion, elbow flexion, elbow extension, wrist palmar flexion, and wrist dorsi flexion. Measurements were taken using a goniometer (stainless steel 300 mm type, University of Tokyo) with participants being in a seated position. Measurements were taken by two physiotherapists. One physiotherapist measured the upper-extremity motions of the participant, while a second physiotherapist ensured the stabilization and positioning of the joint.

### Body Composition Measures

Dual-energy X-ray absorptiometry (DEXA) is established as a conventional method in the evaluation of anthropometry (Mazess et al., 1990; Buckinx et al., 2015). Recently, bioelectrical impedance analysis (BIA) has been used for measuring body composition due to its similarity to DEXA, as demonstrated by several tests (Heymsfield et al., 2005). By sending a weak current through the body, BIA can record reliable and non-invasive measurements. Due to its ease of use in measuring various types of body composition, it has been used in national surveys of large-scale epidemiological studies and in clinical settings (Roubenoff, 1996). In performing a BIA analysis, we used the InBody S10 (Biospace Company Limited, Seoul, Korea) to measure fundamental human body composition: body weight (kg), body muscle mass (kg), total body protein (kg), body mass index: BMI (kg/m^2^), skeletal muscle mass index: SMI (kg/m^2^), body extracellular water ratio: ECW/TBW, dominant hand muscle mass (kg), and dominant hand ECW/TBW. Incidentally, even if the muscle mass of the upper limb does not decrease, SMI decreases when the muscle mass of the lower limbs and trunk decreases (Iwasa et al., 2014). In our study, arm skeletal muscle mass: arm SMM (kg/m^2^), was calculated to follow the change in muscular volume of the dominant hand.

Since InBody S10 allows the measurement to be performed in any position, the measurements were performed while lying down or in a wheelchair, depending on the participant’s condition. The four electrodes 8-point touch electrode method was used by wetting the area where the eight electrodes were attached (thumb and middle finger of both hands, and one on each side of both ankles) with the electrolyte structure and the holder electrode being connected. Although it was difficult for the participants to remain in the position for several minutes to take the measurement, their body positions were adjusted as necessary to the maximum extent possible.

### Analysis

The primary outcome measure was cognitive function since physical activity with upper limb training was expected to improve the same. Physical exercise has been found to improve cognitive function, even among patients with dementia (Pitkälä et al., 2013; de Andrade et al., 2013; Frederiksen et al., 2014). To check group differences, we calculated score changes (post-intervention score minus pre-intervention score) for all cognitive functions, mood state, motor, and body composition measures. We conducted an analysis of covariance (ANCOVA) with permutation tests for each score change, because it is applicable to small sample analysis and corrects for the occurrence of false positives. An ANCOVA with permutation test was conducted to determine significant differences in score changes between the intervention and control groups. Therefore, attending or not attending the DCP intervention was the independent variable, and score changes were the dependent variable. Several covariates could have affected the outcome, including cognitive and psychological measures at baseline, sex, and age. Moreover, motor function and body composition measures affect body size; therefore, motor function and body composition measures at baseline, sex, age, height, and weight were also included as covariates. The “aovp” function of the lmPerm package was used for all ANCOVAs with permutation tests (Wheeler et al., 2016). As permutation indicates significance when the number of samples is limited (Wheeler, 2016), it is suitable for verifying the effectiveness of intervention tests with a small sample size (Nouchi et al., 2013, 2019; Kulason et al., 2018). Moreover, effect sizes (*η*^2^) were calculated by the sum of squares between the groups and the sum of squares by the permutation test of ANCOVA with eta squared (*η*^2^; Cohen, 1988). We then used Storey’s false discovery rate (FDR) correction methods to adjust the *p*-values (Storey, 2002; Table 2). Missing values were not included in the analyses. Statistical significance was set at *p* < 0.05, and all analyses were conducted with R version 3.4.3 (R Core Development Team, 2018, Vienna, Austria).

## Results

During the 3-month period (36 sessions total), 39 of the 46 participants completed all of the measures and training protocol, while five participants from the intervention group and two participants from the control group discontinued their participation in the study. The reasons for drop-out were as follows: less than 60% participation rate (four intervention group participants), hospitalization (one intervention group participant and one control group participant), and death (one control group participant). Therefore, the results of 22 out of 27 members of the intervention group and 17 out of 19 members of the control group were included in the analyses (Figure 2).

### Cognitive Function Results

To measure the effects of DCP on neuropsychological testing, we performed ANCOVAs with permutation tests and FDR to assess changes in both total MMSE-J score and total FAB score. The pre-intervention scores and score changes for each subscore of the MMSE-J and FAB are presented in Table 2. We found significant improvements in the MMSE-J total score in the intervention group (mean = 2.05, SD = 3.98) compared with the control group (mean = −3.24, SD = 4.87; *F*_(1,34)_ = 19.205, adjusted *p*-value = 0.004, *η*^2^ = 0.36; Table 2). Furthermore, there was a significant difference in the total FAB score between the two groups (intervention mean = 2.36, SD = 3.35 vs. control mean = −0.35, SD = 2.15; *F*_(1,34)_ = 8.080, adjusted *p*-value = 0.043, *η*^2^ = 0.19).

We observed significant group differences in MMSE-J subscores for the “spell backwards in reverse” test, with the intervention group showing improved scores (intervention mean = 0.05, SD = 1.70 vs. control mean = −1.06, SD = 1.52; *F*_(1,34)_ = 12.889, adjusted *p*-value = 0.018, *η*^2^ = 0.27). Moreover, for “Language: 3-stage command” scores, there was a significant improvement in the intervention group (mean = 0.41, SD = 1.05) compared with the control group (mean = −0.47, SD = 1.50; *F*_(1,34)_ = 8.006, adjusted *p*-value = 0.028, *η*^2^ = 0.19). Additionally, there was a significant difference in improvement in the intervention group indicated, by an increase in “Recall” scores (mean = 0.50, SD = 0.96), compared with the control group (mean = −0.41, SD = 0.94; *F*_(1,34)_ = 5.932, adjusted *p*-value = 0.047, *η*^2^ = 0.15).

However, there was no significant difference in MMSE-J subscores for “Orientation” between the two groups (intervention mean = 0.50, SD = 1.95 vs. control mean = −0.53, SD = 1.77; *F*_(1,34)_ = 4.734, adjusted *p*-value = 0.075, *η*^2^ = 0.12). There was also no significant difference in MMSE-J subscores for “Registration” (intervention mean = 0.32, SD = 0.95 vs. control mean = −0.47, SD = 1.28; *F*_(1,34)_ = 3.791, adjusted *p*-value = 0.121, *η*^2^ = 0.10); for “Language: names” (intervention mean = 0.05, SD = 0.90 vs. control mean = −0.35, SD = 0.86; *F*_(1,34)_ = 5.603, adjusted *p*-value = 0.060, *η*^2^ = 0.14); for “Language: repeat” (intervention mean = 0.09, SD = 0.43 vs. control mean = 0.12, SD = 0.78; *F*_(1,34)_ = 0.446 adjusted *p*-value = 0.892, *η*^2^ = 0.01); for “Language: following a written command” (intervention mean = 0.14, SD = 0.47 vs. control mean = 0.00, SD = 0.35; *F*_(1,34)_ = 1.035, adjusted *p*-value = 0.621, *η*^2^ = 0.03); for “Language: writing a sentence” (intervention mean = 0.00, SD = 0.53 vs. control mean = −0.18, SD = 0.53; *F* (1, 34 = 3.853, adjusted *p*-value = 0.199, *η*^2^ = 0.10); and for “Visuospatial abilities: copying a diagram” (intervention mean = 0.00, SD = 0.62 vs. control mean = −0.06, SD = 0.43; *F*_(1,34)_ = 0.136, adjusted *p*-value = 0.892, *η*^2^ = 0.00).

Regarding FAB subscores, we observed significant group difference for Go No-Go (inhibitory control). The intervention group showed improved scores (intervention mean = 0.41, SD = 1.10 vs. control mean = −0.18, SD = 0.39; *F*_(1,34)_ = 6.848, adjusted *p*-value = 0.043, *η*^2^ = 0.17).

However, there was no significant difference in the FAB subscores of the intervention and control groups for “Similarities (conceptualization)” (intervention mean = 1.05, SD = 1.25 vs. control mean = 0.76, SD = 1.15; *F*_(1,34)_ = 0.243, adjusted *p*-value = 0.892, *η*^2^ = 0.01); “Lexical fluency (mental flexibility)” (intervention mean = 0.05, SD = 0.58 vs. control mean = 0.06, SD = 0.97; *F*_(1,34)_ = 0.532, adjusted *p*-value = 0.894, *η*^2^ = 0.02); “Motor series (programming)” (intervention mean = 0.55, SD = 1.44 vs. control mean = −0.35, SD = 1.17; *F*_(1,34)_ = 3.268, adjusted *p*-value = 0.111, *η*^2^ = 0.09); “Conflicting Instructions (sensitivity to interference)” (intervention mean = 0.14, SD = 0.99 vs. control mean = −0.47, SD = 0.94; *F*_(1,34)_ = 46.402, adjusted *p*-value = 0.060, *η*^2^ = 0.16); and “prehension behavior (environmental autonomy)” (intervention mean = 0.23, SD = 0.87 vs. control mean = 0.00, SD = 0.71; *F*_(1,34)_ = 0.076, adjusted *p*-value = 0.864, *η*^2^ = 0.00).

These results indicate that cognitive function, which was the primary outcome measure, improved the MMSE and FAB scores. These subscores showed that the “spell backwards in reverse” subscores, “Language: 3 stage command” subscores, and “Recall” scores of MMSE improved. The Go No-Go (inhibitory control) of FAB also expressed improvement.

### Mood State Results

There was no significant difference between the mood state measures of the participants before and after the intervention (Table 2). There was also no significant difference in the GDS-15 scores (intervention mean = −0.41, SD = 2.87 vs. control mean = 0.06, SD = 3.03; *F*_(1,34)_ = 0.481, adjusted *p*-value = 0.864, *η*^2^ = 0.01) and LSI-K scores (intervention mean = 0.32, SD = 2.44 vs. control mean = 0.18, SD = 1.88; *F*_(1,34)_ = 1.869, adjusted *p*-value = 0.336, *η*^2^ = 0.05) of the intervention and control groups.

### Motor Function Results

Both groups exhibited improvements in the handgrip strength of the dominant hand post-training, but there was no statistically significant difference (intervention mean = 0.66, SD = 2.62 vs. control mean = 0.44, SD = 2.51; *F*_(1,34)_ = 1.488, adjusted *p*-value = 0.363, *η*^2^ = 0.04).

We observed improved active range of motion in the wrist palmar flexion of the dominant hand in the intervention group (mean = 10.91 SD = 35.17) compared with the control group (mean = −1.47, SD = 11.56; *F*_(1,34)_ = 16.605, adjusted *p*-value = 0.000, *η*^2^ = 0.33; Table 2). Moreover, the intervention group showed a greater increase in the active range of motion of the shoulder flexion of the dominant hand (mean = 6.14, SD = 28.37) compared with the control group (mean = −5.88, SD = 14.92; *F*_(1,34)_ = 5.887, adjusted *p*-value = 0.040, *η*^2^ = 0.15; Table 2).

However, there was no significant difference in the active range of motion of the elbow flexion of dominant hand between the intervention and control groups (intervention mean = −1.82, SD = 8.10 vs. control mean = −6.47, SD = 21.85; *F*_(1,34)_ = 0.333, adjusted *p*-value = 1.000, *η*^2^ = 0.01). Furthermore, there was no significant difference in the active range of motion of the elbow extension of the dominant hand (intervention mean = 3.41, SD = 14.01 vs. control mean = 2.35, SD = 11.06; *F*_(1,34)_ = 0.770, adjusted *p*-value = 1.000, *η*^2^ = 0.02) and active range of motion of the wrist dorsi flexion of the dominant hand (intervention mean = 4.55, SD = 17.92 vs. control mean = 3.53, SD = 11.96; *F*_(1,34)_ = 4.250, adjusted *p*-value = 0.083, *η*^2^ = 0.11) between the two groups. Ultimately, the results indicate that the DCP led to improvements in the active upper limb range of motion.

### Body Composition Results

We observed significant changes in the intervention group participants’ body composition measures compared with those of the no-treatment control group. The intervention group exhibited decreases in SMI (intervention mean = −0.20, SD = 0.46 vs. control mean mean = 0.10, SD = 0.32; *F*_(1,32)_ = 8.3336, adjusted *p*-value = 0.028, *η*^2^ = 0.21) and dominant hand muscle mass (intervention mean = 0.00, SD = 0.13 vs. control mean = 0.08, SD = 0.18; *F*_(1,32)_ = 6.438, adjusted *p*-value = 0.042, *η*^2^ = 0.17) compared with the control group. We also observed significant decrements in total body protein in the intervention group (mean = −0.10, SD = 0.37) compared with the control group (mean = 0.14, SD = 0.27; *F*_(1,32)_ = 8.117, adjusted *p*-value = 0.028, *η*^2^ = 0.20; Table 2).

Upon weighing both groups, we did not identify any significant group difference in body weight (intervention mean = −0.38, SD = 2.11 vs. control mean = −0.34, SD = 2.28; *F*_(1,33)_ = 0.021, adjusted *p*-value = 1.000, *η*^2^ = 0.00), BMM (intervention mean = −0.57, SD = 1.83 vs. control mean = 0.59, SD = 1.25; *F*_(1,32)_ = 8.998, adjusted *p*-value = 0.083, *η*^2^ = 0.22), BMI (intervention mean = −0.14, SD = 1.03 vs. control mean = −0.22, SD = 1.22; *F*_(1,32)_ = 0.009, adjusted *p*-value = 1.000, *η*^2^ = 0.00), body extracellular water ratio: ECW/TBW (intervention mean = 0.00, SD = 0.01 vs. control mean = 0.00, SD = 0.01; *F*_(1,32)_ = 0.356, adjusted *p*-value = 0.930, *η*^2^ = 0.01), dominant hand ECW/TBW (intervention mean = 0.00, SD = 0.01 vs. control mean = 0.00, SD = 0.01; *F*_(1,32)_ = 1.548, adjusted *p*-value = 0.864, *η*^2^ = 0.05), and arm SMM (intervention mean = −0.01, SD = 0.13 vs. control mean = 0.05, SD = 0.14; *F*_(1,32)_ = 5.100, adjusted *p*-value = 0.089, *η*^2^ = 0.14; Table 2). These results demonstrate that DCP led to the worsening of the muscle composition of the exercised upper limb.

## Discussion

Using a DCP for 3 months, we investigated the effects of physical training of the upper limbs on the cognitive and physical functions of residents of a special nursing home. The participants’ total MMSE and FAB scores indicated that cognitive function improved in the intervention group as compared to the control group. In terms of the subscores, the “spell backwards in reverse” test scores of the “Attention and Calculation” subscore, “Language: 3 stage command” subscores, and “Recall” subscores of the MMSE-J improved, as did the Go No-Go (inhibitory control) subscore of the FAB. Similarly, the upper limb motion range and physical function of the intervention group improved compared to the control group. The active range of motion of the wrist palmar flexion and the shoulder flexion of the dominant hand also improved for the intervention group. Finally, the body composition of the intervention group changed more compared to the control group; the SMI (kg/m 2), total body protein (kg), and the dominant hand muscle mass of the intervention group decreased. We discuss these main findings separately in the following section.

The first main finding was the significant improvement of the total MMSE-J and FAB scores of cognitive functions of the intervention group compared with the control group. The causal relationship between cognitive function and physical function decline was bi-directional in healthy participants (Laurin et al., 2001; Rockwood and Middleton, 2007; Auyeung et al., 2008). Recent studies on scores below MMSE cut-off scores and cognitive impairment offer consistent findings on improving or maintaining cognitive function through exercise interventions (van de Winckel et al., 2004; Kemoun et al., 2010; Venturelli et al., 2011; Vreugdenhil et al., 2012). The risk of dementia continuously reduces with mild physical activity (Byun et al., 2014) and regular physical exercise, even among those with dementia onset (Pitkälä et al., 2013; de Andrade et al., 2013; Frederiksen et al., 2014). In patients with mild to moderate AD, previous studies have reported that walking exercise training has resulted in improved physical and cognitive function (Heyn et al., 2004; Santana-Sosa et al., 2008; Pitkälä et al., 2013; de Andrade et al., 2013; Frederiksen et al., 2014). In a previous study on music-based exercise intervention, baseline mean scores of MMSE were 12.88 (SD = 5.01) in the intervention group and 10.81 (SD = 5.01) in the control group. After 3 months, the score changed to 2.679 (SD = 1.88) in the intervention group and 0.29 (SD = 2.87) in the control group (van de Winckel et al., 2004). These findings indicate that music-based exercise can improve progressive cognitive function in those with lower MMSE scores. Even participants with low cognitive function and low activity can improve cognitive function because they gain the effects of exercise through drumming exercises.

We also observed increases in MMSE-J subscores for Recall in the DCP intervention group [effect size (*η*^2^) was 0.15]. This task uses a delayed language recall task after 2–3 min, as it is known that the hippocampus is important for brain function corresponding to memory domains (Mortimer et al., 2004). We found significant increases in MMSE-J subscores for the spell backwards in reverse test in the DCP intervention group [effect size (*η*^2^) was 0.27]. This task pertains to the attention and concentration domains that may play a role in ADL difficulties (Henneges et al., 2016). We observed increases in MMSE-J subscores for Language: 3 stage command in the DCP intervention group [effect size (*η*^2^) was 0.19]. This task pertains to the comprehension and executive function domains (Trzepacz et al., 2015). Additionally, multimodal exercise tasks and cognitive tasks—combined as dual-tasks—were performed three times per week for 3 months. MMSE and FAB scores showed a significant improvement in the intervention group (Ferreira et al., 2017). FAB scores may have improved because exercise demonstrations require attention and abstraction, continuous execution requires motor sequences, and task persistence requires self-control (De Melo Coelho et al., 2012). In complex exercises, switching and planning exercises requires executive functioning and processing speed; thus, even a short period of exercise intervention could improve global cognitive functioning (Nouchi et al., 2014). Engaging in the DCP program includes the action of stopping and starting, rushing and slowing down, and making minute and loud sounds using drums. Therefore, it can be inferred that frontal cognitive function activity was required and trained through the DCP intervention. We observed significant increases in FAB subscores for Go No-Go (inhibitory control) in the DCP intervention group. The inhibitory control task induces a false-alarm motor response such that the participant should inhibit inappropriate responses (Dubois et al., 2000). Response inhibition is required for accurate hitting timing and the high levels of executive control required for drumming. In particular, music training can lead to gaining more strengthened inhibitory control abilities (Moreno and Farzan, 2015) because of the more robust domain-independent transfer effects that it offers (Schellenberg, 2004). Thus, our results suggest that the drumming program is an intervention method that provides continuous exercise for improving the various cognitive and physical functions of the elderly in nursing homes.

**Figure 3:**
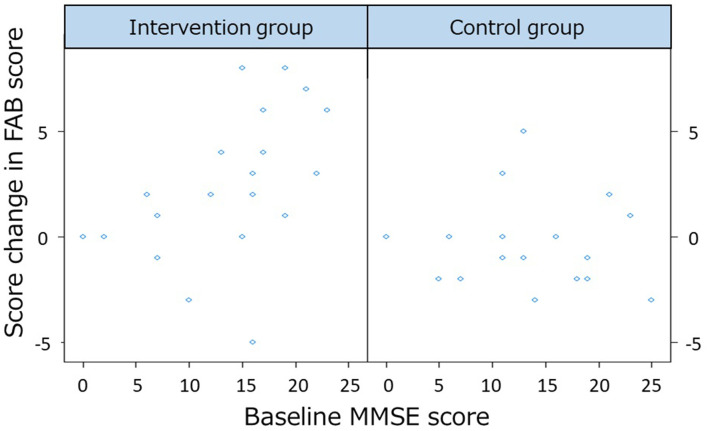
The differences between the improvement of the baseline Mini-Mental State Examination-Japanese (MMSE-J) scores and change in frontal assessment battery (FAB) scores.

The second main finding is that the drumming program provided an opportunity for continued exercise for older people who need a high level of care at a special elderly nursing home. At baseline, compared to previous studies using exercise to improve cognitive functioning (Forbes et al., 2015; Sanders et al., 2019), our participants had the lowest mean score of total MMSE-J (intervention mean = 12.93, SD = 6.64 vs. control mean = 12.89, SD = 6.89). Additionally, intervention activities in many of these previous studies utilized walking, treadmills, and bicycle exercises. Given that our participants had a wheelchair usage rate of 84.78%, they were not expected to engage in whole-body or lower limb exercise. Our participants could train only the dominant hand’s upper limbs while drumming. During exercise training, patients with dementia have difficulty imitating commands and gestures (Della Sala et al., 1987; Vuorinen et al., 2000), but they retain rhythm skills and exercise-induced by rhythm (Vasionyte and Madison, 2013). Even patients with severe dementia exhibit a rhythmic response function (York, 1994, 2000), and exercise with drums is induced by utilizing retained rhythm skills (Vasionyte and Madison, 2013). Rhythm stimulus, such as RAS, entrains the motor system to its beat, which is known for providing walking assistance for Parkinson’s disease. Similarly, the effects of RAS sound are a guiding goal for the participants (Whitall et al., 2000). Target setting and cueing as a guiding goal by rhythm is an important factor for promoting motor learning (Locke and Bryan, 1966). By using the rhythm response function of their remaining upper limbs, it was suggested that participants could play the drums for 3 months, regardless of their degree of dementia. We performed an additional permutation multiple regression analysis to examine the differences between the improvement of the baseline MMSE-J scores and that of the FAB scores. In this analysis, the dependent variable was the change in FAB score, and the explanatory variables were the intervention group, the baseline MMSE-J score, and its interaction term. Age and gender were included as covariates. The lmp function of R was used for the analysis (Wheeler et al., 2016). The results revealed an interaction between the intervention and baseline MMSE-J scores (*F*_(6,32)_ = 4.19, *p*-value = 0.036). The interaction results are plotted in Figure 3. The results suggest that the FAB scores differed from the baseline MMSE scores only in the intervention group. Therefore, higher baseline MMSE scores were associated with higher FAB score owing to the interventional effects of DCP. We recommend intervening before the dementia becomes severe.

The third main finding is that the DCP program effected the body composition of the intervention group, leading to decreased SMI, total body protein, and dominant hand muscle mass. Drumming that requires the use of upper limb muscles while seated is rated as a 3 MET intensity level of physical activity (Miranda et al., 2017), which is classified as equivalent to a light metabolic value. It is a very light exercise for healthy people, but perhaps it has a greater burden on our participants who have lower physical and motor function and live at a special nursing home. Previous studies have shown that low BMI and weight loss are problems among nursing home residents (Blaum et al., 1995; Huffman, 2002; Shen et al., 2019). In the present study, we did not verify malnutrition, but clearly, all participants had sarcopenia at baseline [SMI (kg/m2) intervention mean = 5.01, SD = 1.02 vs. control mean = 4.61, SD = 1.21]. Sarcopenia is characterized by involuntary muscle loss. In younger adults, there is a balance in the preservation of muscle mass between muscle protein synthesis (MPS) and muscle protein degradation (MPD). However, in older adults, the MPS response to dietary protein intake is impaired (Breen and Phillips, 2011). Such anabolic resistance is considered to be an important factor in the etiology of sarcopenia (Farshidfar et al., 2015). The continuous physical activity of drumming for 30 min required the use of the dominant upper limb muscles. Therefore, muscle wasting occurs due to upper limb exercise, and because MPD exceeds MPS, there is a high possibility that muscle mass and total body protein decreased due to exercise load from the drum exercise. Muscle weakness is observed in the early stages of exercise (Hawke and Geary, 2001). Since the participants were older and had a rise in levels of interleukin-6 (IL-6; Ershler and Keller, 2000), or had dementia (Nourhashémi et al., 2002), muscle degradation was increased and muscle synthesis was decreased in response to the exercise load.

To solve this problem, it is important to consume an adequate amount of amino acids to elevate the rates of MPS, while participating in a DCP with exercise effects. Even in older adults, concurrent interventions of dietary protein ingestion and exercise may prevent or slow sarcopenic muscle loss (Breen and Phillips, 2011). In exercise programs at nursing homes, rehabilitation nutrition can be used to improve functionality (Oka et al., 2014). Therefore, it is possible that the body composition changes may have occurred due to lack of nutrition among our participants.

The fourth main finding is that the intervention group showed improvements in their shoulder and wrist flexion of the dominant hand for upper limb motor functions. Particularly, the effect size (*η*^2^) of 0.33 indicated that the intervention group had improvements in active wrist palmar flexion. Task specificity combined with exercise repetition is an important factor for the rehabilitation of the upper limb, which demonstrates improvements in functional mobility and motor strength (Whitall et al., 2000; Winstein et al., 2004). Repetitive bilateral upper limb training with rhythmic cueing was performed on upper extremity patients who had a stroke. Improvements were found in the active range of motion of the shoulder and wrist flexion of the paretic arm (Whitall et al., 2000). Drumming supports the theory of task specificity combined exercise with iteration (Whitall et al., 2000; Winstein et al., 2004) for improving upper limb function to dementia patients and other patients with the debilitating disease of the rehabilitation program.

In addition, participants in the present study were residents of a special nursing home, including bedridden patients. Participants were able to join in the program if they were able to sit for 30 min. The study of upper limb exercise training for patients with diseases that cause weakness is similar to that for patients with chronic obstructive pulmonary disease (COPD), which is a disease that makes it difficult to move the upper limb due to breathlessness and early fatigue of upper limbs. Previously, patients with COPD have performed an unsupported upper limb training program and have shown improvements in physical functioning with or without lower limb training (Ries et al., 2007; Costi et al., 2009; Pan et al., 2012; McKeough et al., 2016). After hitting the drum with a bouncing mallet, the upper limb movement does not support the weight of the arm. In extending our findings, we recommend the use of drumming for COPD patients as well. Upper limb motor function decline impairs the performance of many daily activities such as dressing, bathing, self-care, and writing, which reduces functional independence. Previous studies have reported that residents of nursing homes have poor arm movement (Ostwald et al., 1989) and their poor hand motor function was associated with declined ADL, an increase in functional dependency, admission to a nursing home, and even death (Scherder et al., 2008). Therefore, the upper limb movement and range problem are deeply associated with everyday life.

In this study, the control group showed 3.24 points reduction from the baseline of MMSE scores only for 3 months. Rapid cognitive decline in elderly patients with AD was defined as a loss of 3 or more points in MMSE per year (Barbe et al., 2016). The greatest decline in people with various forms of dementia assessed in a longitudinal study was −4.9 points for 1 year for AD at 65 ± 8 years (Smits et al., 2015). MMSE scores decreased by more than five points in 31% of in-patients with mild to moderate AD at a mean age of 73 years (Musicco et al., 2009). Rapid cognitive decline occurred in 40.9% patients with mild to moderate AD at 80.8 ± 9.0 years (Tchalla et al., 2018). MMSE score decreased from 12 ± 2 at baseline to 6 ± 2 after 6 months in persons with AD aged 84 ± 5 years (Venturelli et al., 2011). Our participants were much older, aged 87.04 (SD = 6.72) years, and their MMSE-J scores were lower (12.91 (SD = 6.67) points) than previous studies. In Addition, immediate family members have a protective effect against rapid cognitive decline (Barbe et al., 2016), however, our participants were institutionalized with the absence of immediate family members. Thus, it is suggested that large cognitive changes may occur due to various factors other than intervention effects.

**Figure 4:**
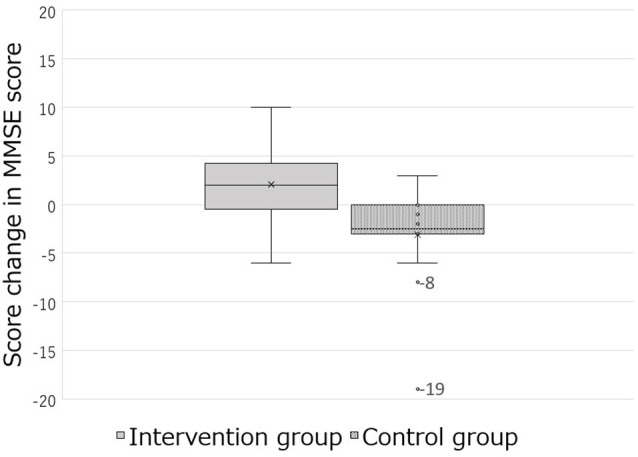
The box-and-whisker plot of the change in MMSE-J score with different interventions.

Therefore, two participants who showed 0% and 100% of the quantile values of MMSE-J change score were excluded from the analysis (Table 3). Furthermore, a total of four participants were excluded from the analysis: two participants who were outliers of MMSE-J change scores in the control group and two who improved in the intervention group (Figure 4; Table 4).

**Table 3:** Comparison of score changes between the intervention and control groups following the DCP intervention (excluding data of two participants whose MMSE-J change scores corresponded to 0% and 100% of the quantile values).

	Intervention group (*n* = 21)		Control group (*n* = 16)		Permutation test *p*-value	Adjusted by FDR *p*-value		Effect size (*η*^2^)
	Mean	SD	Mean	SD				
**Cognitive function measures**						
MMSE-J total score	1.67	3.65	−2.25	2.77	0.000	0.000*	DCP > NT	0.38
Orientation	0.52	1.99	−0.31	1.58	0.042	0.089	ns	0.12
Registration	0.19	0.75	−0.50	1.32	0.083	0.139	ns	0.09
Spell backward in reverse	0.05	1.70	−0.81	1.17	0.000	0.000*	DCP > NT	0.28
Recall	0.52	0.98	−0.31	0.87	0.012	0.034*	DCP > NT	0.15
Language: names	−0.05	0.80	−0.25	0.77	0.070	0.129	ns	0.08
Language: repeat	0.05	0.28	0.19	0.75	0.726	0.841	ns	0.00
Language: 3 stage command	0.29	0.90	−0.31	1.40	0.005	0.029*	DCP > NT	0.18
Language: following a written command	0.10	0.44	0.06	0.25	0.726	0.841	ns	0.00
Language: writing a sentence	0.00	0.55	−0.19	0.54	0.035	0.082	ns	0.11
Visuospatial abilities: copying a diagram	0.00	0.63	0.00	0.37	0.439	0.590	ns	0.01
FAB total score	2.48	3.39	−0.44	2.19	0.003	0.029*	DCP > NT	0.22
Similarities (conceptualization)	1.10	1.26	0.75	1.18	0.388	0.565	ns	0.02
Lexical fluency (mental flexibility)	0.05	0.59	0.06	1.00	0.533	0.691	ns	0.02
Motor series (programming)	0.57	1.47	−0.38	1.20	0.046	0.089	ns	0.10
Conflicting instructions (sensitivity to interference)	0.14	1.01	−0.50	0.97	0.007	0.035*	DCP > NT	0.17
Go–No Go (inhibitory control)	0.43	1.12	−0.19	0.40	0.005	0.029*	DCP > NT	0.19
Prehension behavior (environmental autonomy)	0.24	0.89	0.00	0.73	1.000	1.000	ns	0.00
**Mood state measures**
GDS (Score)	−0.19	2.75	0.31	2.94	0.619	0.774	ns	0.02
LSI-K (Score)	0.24	2.47	0.12	1.93	0.427	0.590	ns	0.05
**Motor function measures**
Hand grip strength (in kg)	0.30	2.04	0.30	2.52	0.327	0.520	ns	0.02
Active shoulder flexion (°)	5.24	28.74	−5.94	15.41	0.005	0.027*	DCP > NT	0.15
Active elbow flexion (°)	−0.95	7.18	−6.88	22.50	0.784	0.858	ns	0.02
Active elbow extension (°)	2.62	13.84	2.19	11.40	0.375	0.565	ns	0.02
Active wrist palmar flexion (°)	14.29	32.18	−2.19	11.54	0.000	0.000*	DCP > NT	0.36
Active wrist dorsiflexion (°)	4.55	17.92	4.38	11.81	0.034	0.082	ns	0.14
**Body composition measures**
Body weight (kg)	−0.28	2.11	−0.46	2.30	0.745	0.841	ns	0.00
Body muscle mass (kg)	−0.51	1.86	0.51	1.24	0.008	0.035*	DCP < NT	0.19
Body ECW/TBW	0.00	0.01	0.00	0.01	0.843	0.868	ns	0.00
Protein (kg)	−0.10	0.38	0.12	0.27	0.010	0.034*	DCP < NT	0.17
BMI (kg/m^2^)	−0.09	1.02	−0.29	1.23	0.824	0.868	ns	0.00
SMI (kg/m^2^)	−0.17	0.45	0.08	0.32	0.011	0.041*	DCP < NT	0.18
Dominant hand muscle mass (kg)	0.00	0.13	0.07	0.19	0.013	0.034*	DCP < NT	0.15
Dominant hand ECW/TBW	0.00	0.01	0.00	0.01	0.083	0.139	ns	0.08
Arm SMM (kg/m^2^)	−0.01	0.13	0.04	0.14	0.044	0.089	ns	0.11

*p < 0.05. SD, standard deviation; Score changes, post score minus pre score; MMSE-J, Mini-Mental State Examination-Japanese; FAB, Frontal Assessment Battery at Bedside; GDS, Geriatric Depression Scale; LSI-K, Life Satisfaction Index-K; Body ECW/TBW, body extracellular water ratio; Protein, total body protein; BMI, body mass index; SMI, skeletal muscle mass index; Arm SMM; arm skeletal muscle mass.

**Table 4:** Comparison of score changes between the intervention and control groups following the DCP intervention (excluding participants who were outliers in the control group and two who improved the most in the intervention group based on the MMSE-J change score).

	Intervention group (*n* = 20)		Control group (*n* = 15)		Permutation test *p*-value	Adjusted by FDR *p*-value		Effect size (*η*^2^)
	Mean	SD	Mean	SD				
**Cognitive function measures**						
MMSE-J total score	1.30	3.33	−1.87	2.39	0.000	0.000*	DCP > NT	0.33
Orientation	0.35	1.87	−0.40	1.59	0.042	0.098	ns	0.10
Registration	0.20	0.77	−0.33	1.18	0.235	0.391	ns	0.07
Spell backward in reverse	0.05	1.79	−0.87	1.19	0.000	0.000*	DCP > NT	0.31
Recall	0.55	1.00	−0.33	0.90	0.013	0.048*	DCP > NT	0.16
Language: names	−0.15	0.67	−0.13	0.64	0.548	0.707	ns	0.01
Language: repeat	0.10	0.31	0.27	0.70	0.824	0.873	ns	0.00
Language: 3 stage command	0.15	0.67	−0.13	1.25	0.017	0.054	ns	0.14
Language: following a written command	0.05	0.39	0.07	0.26	1.000	1.000	ns	0.00
Language: writing a sentence	0.00	0.56	−0.20	0.56	0.045	0.099	ns	0.12
Visuospatial abilities: copying a diagram	0.00	0.65	0.00	0.38	0.594	0.707	ns	0.01
FAB total score	2.60	3.42	−0.47	2.26	0.007	0.034*	DCP > NT	0.23
Similarities (conceptualization)	1.20	1.20	0.80	1.21	0.396	0.578	ns	0.02
Lexical fluency (mental flexibility)	0.05	0.60	0.07	1.03	0.592	0.707	ns	0.02
Motor series (programming)	0.55	1.50	−0.50	1.24	0.069	0.133	ns	0.09
Conflicting instructions (sensitivity to interference)	0.15	1.04	−0.53	0.99	0.009	0.039*	DCP > NT	0.18
Go–No Go (inhibitory control)	0.45	1.15	−0.20	0.41	0.003	0.028*	DCP > NT	0.20
Prehension behavior (environmental autonomy)	0.25	0.91	0.00	0.76	0.922	0.949	ns	0.00
**Mood state measures**
GDS (Score)	−0.15	2.81	0.33	3.04	0.421	0.590	ns	0.01
LSI-K (Score)	0.20	2.53	0.13	2.00	0.077	0.142	ns	0.06
**Motor function measures**
Hand grip strength (in kg)	0.28	2.09	0.32	2.61	0.581	0.707	ns	0.01
Active shoulder flexion (°)	3.50	28.34	−6.00	15.95	0.037	0.060	ns	0.12
Active elbow flexion (°)	−0.75	7.30	−7.33	23.21	0.391	0.578	ns	0.02
Active elbow extension (°)	2.00	13.90	2.67	11.63	0.606	0.707	ns	0.01
Active wrist palmar flexion (°)	16.25	31.70	−2.33	11.93	0.000	0.000*	DCP > NT	0.37
Active wrist dorsiflexion (°)	6.50	16.94	4.67	12.17	0.024	0.065	ns	0.14
**Body composition measures**
Body weight (kg)	−0.21	2.15	−0.57	2.33	0.824	0.873	ns	0.00
Body muscle mass (kg)	−0.49	1.90	0.58	1.25	0.005	0.034*	DCP < NT	0.19
Body ECW/TBW	0.00	0.01	0.00	0.01	0.375	0.578	ns	0.02
Protein (kg)	−0.09	0.39	0.13	0.27	0.014	0.048*	DCP < NT	0.18
BMI (kg/m^2^)	−0.05	1.04	−0.35	1.24	0.784	0.873	ns	0.01
SMI (kg/m^2^)	−0.16	0.46	0.10	0.06	0.007	0.034*	DCP < NT	0.21
Dominant hand muscle mass (kg)	0.01	0.13	0.07	0.19	0.022	0.065	ns	0.14
Dominant hand ECW/TBW	0.00	0.01	0.00	0.01	0.231	0.391	ns	0.09
Arm SMM (kg/m^2^)	−0.01	0.13	0.04	0.14	0.053	0.108	ns	0.10

*p < 0.05. SD, standard deviation; Score changes, post score minus pre score; MMSE-J, Mini-Mental State Examination-Japanese; FAB, Frontal Assessment Battery at Bedside; GDS, Geriatric Depression Scale; LSI-K, Life Satisfaction Index-K; Body ECW/TBW, body extracellular water ratio; Protein, total body protein; BMI, body mass index; SMI, skeletal muscle mass index; Arm SMM; arm skeletal muscle mass.

For additional analysis, two participants were excluded; we found significant improvements in the MMSE-J score in the intervention group (mean = 1.67, SD = 3.65) compared to the control group (mean = −2.25, SD = 2.77; *F*_(1,32)_ = 19.531, adjusted *p*-value = 0.000, *η*^2^ = 0.38; Table 3). Furthermore, there was a significant difference in the FAB score between the two groups (intervention mean = 2.48, SD = 3.39 vs. control mean = −0.44, SD = 2.19; *F*_(1,32)_ = 8.874, adjusted *p*-value = 0.029, *η*^2^ = 0.22; Table 3).

Similarly, when the four participants ware excluded, we found significant improvements in the MMSE-J score in the intervention group (mean = 1.30, SD = 3.33) compared to the control group (mean = −1.87, SD = 2.39; *F*_(1,30)_ = 14.809, adjusted *p*-value = 0.000, *η*^2^ = 0.33) and in the total FAB score between the two groups (intervention mean = 2.60, SD = 3.42 vs. control mean = −0.47, SD = 2.26; *F*_(1,30)_ = 9.104, adjusted *p*-value = 0.034, *η*^2^ = 0.23; Table 4).

Therefore, the results of these analyses suggest that the primary outcome measures of cognitive function improved almost as much as the results for all participants (Table 2). Since the permutation test was adopted to the analyses in order to avoid the influences of outliers (Wheeler et al., 2016).The very elderly people, especially institutionalized residents, would show greater decline if they do not recieve treatment (such as the one provided through the intervention in this study).

Our study has some limitations. The 3-month intervention period was a short period, but longer-term interventions may lead to better physical function. Moreover, it is essential to determine the exercise load accurately for exercise intervention studies. The study did not measure the actual exercise load due to drumming. Future studies will measure momentum. Additionally, we did not investigate changes in ADL. Improvement in the shoulder and wrist palmar flexion may have been effective for items such as the meal action of the participant and toilet disposal. ADL intervention studies should also be carried out to assess care burden. Previous studies have shown that social engagement is likely to contribute to the effect of cognitive function improvement (Dawes et al., 2015; Penninkilampi et al., 2018). A study in which this study and the program were similar reported that intervention with group drumming sessions improved the social resilience of patients who were receiving mental health services (Fancourt et al., 2016). In future research, we want to further investigate social engagement. Furthermore, since certain tests, such as the magnetic resonance tests, are expensive, we did not diagnose the specific type of dementia that the participants experienced. Finally, we did not use conduct program for the control group. Given that we wished to identify the differences between the ordinary daily exercises at the nursing home and the DCP exercise effects, we used a waitlist control group.

## Conclusion

This study was designed to investigate the effects on cognitive and physical function of participating in a DCP intervention in older adults with cognitive impairment and dementia living in a special nursing home in Japan. Most of the participants used wheelchairs and had dementia, posing a difficulty in comprehending commands or imitating gestures. However, they were able to participate in the DCP training of the upper limb physical function for 3 months by utilizing their retained rhythmic function. Our results show that drumming movements can provide sufficient physical activity and exercise to improve cognitive function. The body composition of those who participated also changed more when compared to the control group. These results suggest that DCP as an intervention method can be used to promote exercise and improve the various health and cognitive functions in special nursing homes.

## Data Availability Statement

The datasets acquired and/or analyzed during the current study are available from the corresponding author upon reasonable request.

## Ethics Statement

The studies involving human participants were reviewed and approved by The Ethics Committee of RIKEN. The patients/participants provided their written informed consent to participate in this study.

## Author Contributions

AM designed and developed the study protocol and conducted the study. TO, HM, and KS were physiotherapists and evaluated measurements. MI judged the exclusion criteria and screened participants. AM and RN wrote the manuscript. RN provided advice related to whole study. All authors read and approved the final manuscript.

## Conflict of Interest

KS was previously employed by the company Care 21 Co., Ltd. The remaining authors declare that the research was conducted in the absence of any commercial or financial relationships that could be construed as a potential conflict of interest.
